# Barriers to abortion care for recently arrived migrants in Germany

**DOI:** 10.1016/j.jmh.2026.100417

**Published:** 2026-05-19

**Authors:** Talia Meer, Alex Müller, Mascha Kern, Bornice Biomndo, Yagmur Demirpehlivan, Muhammad Helmi Barghouth, Stefanie Theuring

**Affiliations:** a Department of History, Humboldt University of Berlin Germany; b Gender Health and Justice Research Unit, University of Cape Town, South Africa; c Institute of Public Health, Charité – Universitätsmedizin Berlin, corporate member of Freie Universität Berlin and Humboldt Universität zu Berlin, Germany; d Institute of International Health, Center for Global Health, Charité – Universitätsmedizin Berlin, Germany

**Keywords:** SRHR, Abortion, Migrant, Refugee, Reproductive rights, Germany, Qualitative research

## Abstract

In Germany, abortion is criminalised but permitted without punishment through a specific mandatory counselling process. Navigating this highly regulated pathway may be particularly challenging for recently arrived migrants such as asylum-seekers, who often face specific vulnerabilities. The study analyses the barriers to access to abortion for recent migrants in Germany, to identify barriers for recent migrants with unwanted pregnancies by analysing qualitative data from 17 interviews with counsellors providing mandatory counselling, client advocates, and healthcare providers in the federal states of Berlin and Brandenburg. We employed a thematic analysis approach using MaxQDA.

We identified two kinds of barriers for migrant access to abortion care in Germany. The first includes barriers that arise from the current legal frameworks for abortion and for migration, specifically related to mandatory counselling for abortion-seekers in Germany, and housing in collective large-scale accommodation facilities for asylum-seekers. The second relates to health system capacity and includes barriers that directly result from the limited and unequal availability of information and services in the languages spoken by clients. Interviews revealed a clear difference between Brandenburg, where abortion services are limited and predominantly provided by white German-speakers, and Berlin, where there is a more diverse network of healthcare providers, client advocates and support persons, relatively better access to care for migrants and language needs are more systemically addressed.

Our findings of the disproportionate barriers experienced by migrants and asylum-seekers under the current abortion pathway further underscore the importance of the recommendation for decriminalisation of abortion in the first trimester, and of the diversification of healthcare providers. This would ensure better access to abortion care for migrants and asylum-seekers, themselves often a vulnerable group at higher risk for an unwanted pregnancy.

## 1 Introduction

In Germany, abortion is criminalised but permitted without punishment through a specific mandatory counselling process (Strafgesetzbuch StGB § 218, 219. Bundesamt Für Justiz 2024). Abortion is regulated under §218 of the German Penal Code, according to which abortion is illegal but is not punishable if it is performed within the first 12 weeks of pregnancy (with specific exceptions of medical necessity or sexual assault) and if the abortion-seeker has undergone mandatory counselling (Strafgesetzbuch StGB § 218, 219. Bundesamt Für Justiz 2024). Individuals must attend the mandatory counselling at a state-recognized centre or registered gynaecologist. According to the law, the counselling session should be conducted without a pre-determined outcome, yet the same law states that the overarching aim of the mandatory counselling is to ‘protect the unborn life’ (Bundesamt für Justiz 2024). The counselling session includes medical, social and legal information, including about support services available should the abortion seeker decide to continue the pregnancy (Bundesamt für Justiz 2024). Abortion-seekers are issued a counselling certificate, with which, after a mandatory waiting period of three days, they access an abortion with a registered healthcare provider, different from the one who provided the mandatory counselling.

Although most residents in Germany, including documented migrants, are covered by health insurance, it only covers the costs of abortion if it is medically necessary or if the pregnancy resulted from a crime. Hence, most abortions are not covered by health insurance. People typically bear the cost themselves, which can range from €200 to €700 depending on the type of intervention (Schwangerschaftsabbruch, profamilia 2024). Federal programmes cover abortion costs in cases of financial hardship, including for migrants. It is estimated that 60–70% of people seeking abortions make use of this financial support (Maeffert and Tennhardt, 2023).

Navigating this highly regulated pathway may be particularly challenging for recently arrived migrants such as asylum-seekers, who often face specific vulnerabilities. On the one hand, people with refugee experience are at particular risk for unintended or forced pregnancies, sexual violence, and unsafe abortions, both during flight and after arrival in the host country (Cayreyre et al., 2024; Erhardt-Ohren and Lewinger, 2020; Keygnaert and Guieu, 2015; Lehmann, 2002). A study in Germany estimated the unmet need for family planning among asylum-seeking women to be 47% (Inci et al., 2020). On the other hand, international evidence demonstrates that asylum-seekers and recent migrants may have lower knowledge and access to sexual and reproductive healthcare in their new context (Hasstedt et al., 2018; Tirado et al., 2023). Yet, the specific situation of recent migrants, including asylum seekers, seeking abortion in Germany, and globally, remains poorly understood (Erhardt-Ohren and Lewinger, 2020; Inci et al., 2020). In the German healthcare system, bureaucracy, difficulty in navigating care pathways, language, socio-cultural barriers, and negative experiences with healthcare providers have been observed to hinder migrants accessing health services. High illiteracy levels, precarious living conditions, lack of income, health insurance, and documents like proof of identification or residence, are factors that can make migrants fearful of seeking services (Ärzte der Welt 2024; Behrensen and Groß, 2004; Busch et al., 2022; Frings, 2017; Hahn et al., 2020). In a study that analysed requests from Germany for telemedicine abortions by mail from the organisation ‘Women on Web’, 9% of abortion seekers identified as undocumented immigrants, and 21% reported difficulties related to being a migrant as a reason for seeking an abortion outside of the formal German healthcare system (Killinger et al., 2022). Individuals described denial of access to services, language barriers, knowledge gaps about the complex regulatory system, and fear related to their immigration status (Killinger et al., 2022).

This article contributes to the emerging literature on abortion services in Germany, which is still under-researched. Specifically, it contributes to understanding how migration and reproductive healthcare regimes intersect to create barriers to abortion access for recently arrived migrants with unwanted pregnancies, and how mandatory counselling functions as a structural barrier (Meer et al., 2025). Further as we analyse qualitative data from interviews with counsellors providing mandatory counselling, client advocates, and healthcare providers in the federal states of Berlin and Brandenburg, we articulate a view of service provision and constraints from a perspective not yet sufficiently accounted for in the literature. Understanding the barriers to abortion care for migrants and asylum-seekers in Germany is important not only to elucidate the current domestic situation, but also because the nexus of migration and abortion rights sheds light on two globally relevant issues: understanding the barriers to access for migrants in Germany can inform care in multiple other contexts where migration necessitates the adaptation of healthcare provision, and where abortion remains criminalised.

## 2 Methods

### 2.1 Design

We chose a qualitative study design using individual stakeholder interviews to gain in-depth insights into the topic. To compare rural and urban contexts our study focused on the German federal states of Berlin and Brandenburg. Brandenburg is more than 30 times larger than Berlin but sparsely populated and characterized by rural landscapes. Berlin, a city-state, is hugely diverse with almost a quarter of its population being migrants and a well-established migrant service provider network, whilst in Brandenburg only 7.5% of inhabitants are foreigners (Population by nationaly and federal states 2023). However, in recent years, more asylum-seekers are being accommodated in the state, making understanding migrant access to care even more urgent. Berlin also offers comparatively good access to abortion services, with a higher concentration of medical facilities and psychosocial counselling centres than many other federal states, including surrounding Brandenburg (Hahn et al., 2025). For these reasons, Berlin represents a suitable site to study access to abortion and barriers for migrants. By contrast, Brandenburg has fewer doctors providing abortions (Torenz et al., 2023), and services for migrants are also fewer, providing a strong contrast in perspectives. By studying these neighbouring states, we can draw conclusions about how access to abortion differs and connects across states in Germany.

### 2.2 Data collection

Through service-mapping in the two states, we identified all state-registered organisations offering mandatory counselling, and specific healthcare providers offering abortion care. From this list, we invited a range of service providers to interviews about their role and experience. All invitations were extended by email, although follow-up sometimes occurred by telephone, and included detailed study information, information about ethics and informed consent, as well as contact information for both researchers and university ethics committees. In Brandenburg, we additionally purposively recruited providers located in areas with asylum shelters. In both states, snowball sampling was employed following the initial interviews, through which we were referred to further relevant interviewees. Data collection continued in parallel with data analysis until thematic saturation was reached, where additional interviews produced no emerging themes regarding the processes required of migrants seeking abortion and the barriers that they faced. All interviews took place between November 2023 and July 2024. Researchers conducting interviews had no personal connection to study participants.

In total, we conducted 17 semi-structured Interviews with individuals providing abortion-related services before, during and after termination of pregnancy: nine providers of mandatory counselling (including seven social workers and two gynaecologists); one doctor’s assistant at a practice providing abortions with majority migrant client-base; five advocates for abortion access (including one representative each from pro-choice, sex worker, migrant, drug use organisations; and one from a support group for people who have experienced abortion); and two translators deployed in abortion service provision. A greater proportion of people was interviewed in Berlin compared to Brandenburg; this is a result of contacting people that counsellors directly referred to in Berlin, whereas counsellors in Brandenburg did not have a wider network.

Interviews were conducted depending on participant preference, via video call or in-person, in German or English, and lasted between 50 and 90 minutes. At the outset, participants provided written informed consent, either by signing consent forms in person, or emailing digital copies of signed consent forms. Interviews began with information about the offerings of the participants’ organisations and then explored experiences in providing abortion-related care with a focus on barriers that impact recently arrived migrants’ access to care. Interviewers kept fieldnotes.

### 2.3 Data analysis

The qualitative data from the expert interviews were recorded, transcribed, and translated into English when needed. The transcripts were circulated back to the interviewees for validation (Rowlands, 2021). Codes were developed jointly by the research team, reading transcripts for common themes. Based on this initial coding scheme, all transcripts were then coded by one researcher using MaxQDA, and codes were grouped into broader themes (MAXQDA | All-In-One Qualitative and Mixed Methods Data Analysis Tool 2019). The themes were refined in a continuous iterative process and discussed by the research team to produce the final thematic framework.

Where not stated otherwise, the term “migrant” is used to refer to recently arrived migrants, such as asylum-seekers.

The study was approved by the research ethics committee of Charité - Universitätsmedizin Berlin (Vote EA4/152/23, 30th August 2023). Confidentiality and anonymity were ensured to all interviewees, and specific characteristics of the interviewee or her organisation were de-identified where necessary.

## 3 Findings

All 17 interviewees were female; nine were counsellors (four based in Brandenburg, five in Berlin), six were abortion-related support personnel, two worked in translation in healthcare settings, and one was a doctor’s assistant at a practice that provides abortions (all in Berlin, though one also worked in Brandenburg). Ages ranged from 24 to 65, with an average age of 46. Although all of the nine interviewees providing mandatory counselling were perceived as white German-speakers by our team in both Berlin and Brandenburg, in Berlin counsellors were part of a network that was more diverse, with five support persons and translators who identified as migrants.

### 3.1 Legal determinants of abortion access

#### 3.1.1 The legal requirement of mandatory counselling

Although most of our interviewees were counsellors providing mandatory abortion counselling themselves, many expressed scepticism about its role and utility for migrants and asylum-seekers. One translator pointed out that many people come from places where it is easier to get an abortion than in Germany, and “don't understand why they have to get this [counselling] from the social worker” and feel that: “this is my body, I have to decide for myself” (Interview17).

For migrants who already must interface continuously with the complex bureaucracy of the immigration system, the legal requirement of mandatory counselling may be perceived as just another appointment at another office:

The vast majority of people have a lot of appointments with the authorities. At the end of the day, we are also perceived as just an authority. […] Yes, of course, this is mandatory counselling. Of course, we are [another] authority. (Interview05, counsellor, Berlin)

One counsellor said that she sometimes feels that clients see the counselling appointment as having a bearing on their residency application:

I think that women are not able to understand, is this a hearing for my asylum claim, or [what is going on]? Someone comes along, a woman who says that she works at [a counselling organisation] and she can ask [her] questions. I can´t decide anything [about asylum], I only have a counselling function, but I think there’s still fundamental scepticism about what role [counselling] actually plays. What is done with [the] answers? (Interview08, counsellor, Brandenburg)

Another counsellor in Brandenburg also observed that migrant women may be especially reserved during sessions, focusing on the medical information and the goal of getting the certificate that allows them to get an abortion, rather than on the counselling.

It's often the case with foreign women that they really want to be informed about the abortion methods, but they don't want to go into so much depth. That's my experience. They want a counselling certificate. (Interview06, counsellor, Brandenburg)

At the same time, from the perspective of a Berlin-based counsellor, abortion-seekers were often quite grateful for the opportunity to talk about their situation; accordingly, she would not favour the complete abolition of abortion counselling, while acknowledging that the compulsory aspect of it “always sucks” (Interview03).

Moreover, cultural and linguistic differences between counsellors and migrant clients may significantly lower the establishment of trust and rapport. Interviewees repeatedly pointed this out as a barrier:

I know […] especially the Brandenburg colleagues, but also the Berlin colleagues and also other providers. And I have the impression that we counsellors are female, white and generally German. So, I also think that the diversity should somehow be even greater. (Interview10, counsellor, Brandenburg)

Sometimes the questions about the child's father […] were not answered [by the client]. And at some point, I thought, do you just accept that? Because I can't really put myself in this whole life situation. I can empathise, but I don't know what the women have experienced on the run, what happened beforehand. How much trust a woman needs on such a journey. Why should she suddenly have a lot of trust just in me? (Interview08, counsellor, Brandenburg)

[W]hen someone comes into the room for counselling for something like that [abortion], they come with their whole world, you don't see it, you're just treating the person in front of you. But in the head and the decision-making of the person there is a whole society behind that person, and [the counsellor] basically asks you to be here as a white western atheist, no story, best privileged life person (Interview11, advocate, Berlin).

Whilst most recognised this as a structural constraint, some expressed annoyance at their migrant clients. One counsellor explained that she might not always enquire further about concerning issues if language is a barrier, because for migrants “perhaps that is the price to pay”:

Yes, you could say, I could get involved here now, to make it all work, but the question is, there are also, yes, there are unclear things where the women don't express themselves clearly and then there's no interpreter on site for the doctor. You could perhaps say, and this sounds a bit trite, that the woman is helped […] by having the abortion, and she does not get to choose the method and the location. That's perhaps not entirely fair compared to a German woman who can do that, but I would say that's perhaps the price to pay. (Interview08, counsellor, Brandenburg)

Scepticism towards the German mandatory counselling system does not, however, mean that migrants do not have concerns about abortion or do not want to discuss these with someone. One abortion advocate who herself had an abortion in Germany explains:

For me one of the few things that really made me more at peace, was that when someone who was also like a mixed [nationality] and also from a Muslim background, a friend, who's a midwife told me like, ‘Hey you know like it's part of a normal gynaecological life, I don't mean to tell you you're not special, but like you're kind of not special, you´re just having an abortion’, and to hear that from someone who's exactly from the same background from me, and also same religious background and tradition, to hear that from someone like that, it was something that was more relieving than anyone I met in the German system. (Interview11, advocate, Berlin)

#### 3.1.2 The legal requirement of collective temporary accommodation for asylum-seekers

Asylum-seekers are usually housed in large temporary collective accommodation centres. One advocate for migrants who works in these collective accommodation facilities describes the lack of control and agency that people housed in these facilities have, including about their health:

You were super brave to reach [Germany]. [And once they are here], then they're transferred and nobody gives no answer or not any kind of argument, you know, why does it happen, you know? So, you just follow rules, fucking rules, from a bureaucracy that is absolutely invisible and you feel so powerless because this is all your life as a migrant and as a refugee. [State officials unknown to you], like some invisible power, take decisions, existential decisions about you, you know, all your life. (Interview15, advocate, Berlin and Brandenburg)

Interviewees who work in these facilities pointed out that in general, care, including information about sexual and reproductive health, contraception and abortion, is not made available as a matter of course, and is dependent on the initiative of those who work there:

Interviewer: But that means that at the moment they don't offer that [information] in the temporary accommodation centres, did I understand that correctly?

Interviewee: There used to be, but it's no longer there. It used to be there for a while and then it changed because the staff in the transitional homes also changed. It often has to do with people. At that time, there were still refugees who were already working there themselves and they were the links. (Interview09, counsellor, Brandenburg)

But I must say I think that 90% of the possibilities you have in a place depend a lot on the people who are working there. So, in some shelters, the people are there like 20 years and they don't have anything, no energy, no… And some of them are super new and have a lot of energy and they really help people; they really want to do. And I kind of like a new generation who is really involved in this. (Interview15, advocate, Berlin and Brandenburg)

So, to begin with, abortion-seekers must enquire about options, and reveal to an official in the accommodation that they are pregnant and would like to seek an abortion. This may require considerable courage given their vulnerable position, as one counsellor observes:

And it's also a hurdle to talk to strangers about the fact that you are now unintentionally pregnant and want to have an abortion. I can imagine that it's difficult to go to the social worker at the refuge and say that I'm pregnant and want an abortion. (Interview01, counsellor, Berlin).

Some counsellors offer mandatory counselling in temporary accommodation centres. However, due to the way that these centres are organized, they often do not provide an adequate space in which to conduct a confidential counselling session. One counsellor in Brandenburg described how the counselling sessions in one accommodation centre are not optimal for counselling, and how she experiences the chaos of the environment:

Then it might not be as counselling-like as you would imagine in a counselling centre, because experience has shown that especially at times when there was a lot of traffic, there was of course a bit of chaos, well chaos, how should I put it, there are just a lot of people there. […] Then you have a glass door, [and enter the room] where there is a crowd of people and where you have to push through. […] And then, in the best-case scenario, we are let in. Sometimes we knock on the door a bit and aren't let in straight away because they've forgotten us, maybe in the chaos or whatever. (Interview08, counsellor, Brandenburg)

The same counsellor tells of one woman in this accommodation centre, who was scheduled for an abortion, but risked losing her close-knit group, who were being moved to different accommodation before the date of her abortion:

There was a woman who was supposed to go for a transfer and now, as an example, she had found a small group who were all supposed to go for a transfer to [town] and because of the [abortion] this was probably postponed for her and she didn't understand that at all. That really moved her because she said she still wanted to go because she was probably also afraid of losing this small group. […] perhaps the outpatient clinic meant well, like, `we're still going through with the intervention [abortion] because we've now initiated it`, and something completely different was important to the woman at that moment. […] I don't think [the structures] are transparent for the women. Even for us, they are barely understandable. (Interview08, counsellor, Brandenburg)

In this context, the woman prioritized staying with the other asylum-seekers she had formed relationships with rather than getting an abortion sooner, but the officials persisted in “going through with the intervention”.

### 3.2 Health system determinants of abortion access

#### 3.2.1 Delays in receiving health insurance coverage

Asylum-seekers can apply for the coverage of abortion costs through the federal financial hardship provisions. According to the law, they should also have basic health insurance, which is necessary to access a gynaecologist to determine the week of pregnancy and any necessary aftercare. However, in Berlin there have been delays with the issuance of health insurance cards as outlined by one district health officer responsible for uninsured people:

When the women are registered there, a letter is sent to the health insurance company - there is now a huge pile of letters and then some poor person sits there and has to enter it all because this digital transmission no longer works. As a result, they sometimes have to wait three or four months before they even get the letter or a health insurance card. And the doctors' practices outside don't take them if they don't have the letter or the card. (Interview03, gynaecologist and counsellor, Berlin)

This is a crucial barrier to accessing the care necessary to start the process of accessing an abortion.

#### 3.2.2 Availability of information in languages other than German

Among our interviewees, almost all observed language barriers as a stumbling block for migrants, and as shaping their ability to provide services to them. This was noted throughout the abortion pathway and pertained specifically to availability of good-quality information, and the availability of care in the appropriate language or translation services.

Whilst some advocacy groups in Germany have made plain language information available in English, this is less readily available in other languages. Several interviewees pointed out that for new immigrants finding information is a considerable challenge:

Well, I would say language barriers of course, knowledge of the system, then knowing where I can somehow call to organize this conflict counselling, this counselling session somehow, also with language translation, which then also has to be done quite quickly, then also somehow find a doctor or a day clinic for the abortion itself. Navigating all of this on your own. (Interview13, advocate, Berlin)

[There is] so little information. I came as a migrant myself. But I have worked in hospitals. I have knowledge from the internet. And many uneducated women, they can't find [information] easily because of language barriers. My opinion is that this is an important topic […] And you should make big signs somewhere in the subway. That there are these offers [for abortion care]. […] But many people come and they don't know what to do with this pregnancy. They don't know what the law is here in Germany (Interview17, professional translator, Berlin)

Language barriers may continue to be a hindrance along the care pathway. However, the provision of translation is not regulated, and each facility must arrange translation themselves.

For several interviewees, many, if not the majority, of their clients were migrants. Some had in-house translators on specific days for specific languages and relied on on-demand translation services, both informal and from agencies, for other languages. Others depended on state-funded translation services. Yet others had no translation services at all. This was specifically the case in Brandenburg.

In Berlin, the state-funded translation service has a license for medical translations and offers services online and in-person. This has been widely taken up by providers. However, interviewees noted that it is not always possible to select the translator according to their subject competence, or their gender.

You always want female people, but that usually doesn't work out. Sometimes you have an older man as a language mediator, so they have a laptop here, and then we try to have a preliminary discussion with him, but that doesn't always work out. (Interview05, counsellor, Berlin).

Counsellors also noted that clients may be more reserved with translators, and that translators are not always able to set the client at ease:

[Clients are] often not, at least it doesn't seem, that emotional to me in counselling. Probably because [clients] [don’t] want to get it off their chest, perhaps in front of the interpreter. You never know what relationship the interpreter has with the women. That's also an inhibition, so to speak, for the women not to open up quite so much. That's perhaps a bit of a difference to the other [counselling sessions] that take place in our mother tongue. (Interview06, counsellor, Brandenburg)

One translator herself said that she often needs to reassure clients that she is neutral and confidential, and that although she speaks the same language or comes from the same country, she is acting as a professional:

Women must have confidence. Many women come [to us] afterwards, we meet them in the hallway. They ask, do you know anyone from our country? They think I can pass their story on. At the beginning we always tell [them] about data protection, the duty of confidentiality. That's so important. I said, no, we are like doctors, we are subject to confidentiality. (Interview 17, professional translator, Berlin)

In this regard, the cultural and linguistic likeness of the translator creates a sense of familiarity that can be comforting to clients, or that can feel threatening given clients fears of judgement or punishment from community members.

Where services do not provide translators, abortion-seekers are expected to organize someone who can translate to accompany them to appointments. Relying on a relative or a friend may not be possible for those without the necessary social networks, or stigma may prevent people from reaching out. As a result, individuals often rely on partners, raising concerns for counsellors and healthcare providers who have no way to ensure accuracy or neutrality in the translation, as one counsellor describes:

It's in fact mainly just the men of those women who come along, who already have a better knowledge of German and then help translate. […] Ultimately, I don't know whether he will really translate what I ask him, so I can only trust him. Yes, and whether he will also correctly translate what she then says. It's not a neutral translator. (Interview06, counsellor, Brandenburg)

The same counsellor notes that in the absence of alternatives, children may also be relied on for translation. However, counselling centres increasingly do not permit this:

We used to have a time when they came here with the children, with the older children, who were supposed to interpret the counselling, and we put a stop to that. […] So, this really is a neutral place where they really have peace and quiet to talk about it. (Interview06, counsellor, Brandenburg).

If the abortion-seekers cannot bring a person who can translate during the appointments, some counselling centres or medical practices may use Google Translate:

I was just talking to a doctor about it yesterday and he said we don't have the capacity to organise [translators]. If the patients don't come with a friend, relative or whoever, then we'll use Google Translate or something like that. (Interview01, counsellor, Berlin).

Other providers also mentioned relying on translation apps, however, as one provider illustrates, with specific reproductive health terminology, or with linguistically specific euphemisms frequently used to discuss stigmatised topics, this “is sometimes a disaster”:

When we have Google translator and we translate Vietnamese, the woman says “My boat hasn't arrived. The bus hasn't travelled. My boat hasn't arrived.” Then I think to myself, okay, that makes no sense. […] Some people write with Google translator: “I would like to come to your workshop for my screw”, it's about the IUD, but that's a problem. (Interview04, counsellor, Berlin)

Worst still some providers are not inclined to resort to such solutions and refuse the service to the abortion-seeker and “if the woman doesn't have someone here to translate”, will “send her away again” (Interview02).

Translation is also time-consuming and costly, with providers asserting that “interpreters simply need double the time for every appointment” (Interview03). This is especially so when individuals have complex needs or especially difficult circumstances, but taking longer with individuals puts pressure on individual organisations who also must meet quotas for client numbers and resource allocation:

[W]e sometimes come under pressure because of the cost performance calculation that we have somehow made […] too little per month, but we can put up with all that. I think it's more the case that we have less turnover, but the individual cases we have are simply much more complicated, take much longer to process. (Interview03, gynaecologist and counsellor, Berlin).

Nevertheless, translation of both online sources and in other informational materials, as well as in counselling and healthcare provision itself, is essential to providing equitable care for migrants and asylum-seekers. Especially in counselling, language is the prerequisite for good quality care:

You couldn't do advisory work without language. How is that supposed to work? I can't paint without colours. So that doesn't work. (Interview09, counsellor, Brandenburg)

However, translators are not interchangeable, and as described by the coordinator in Berlin, finding the right fit, including the gender of the translator, can make all the difference:

And we also get feedback: […] “We would like the same colleague again for this assignment and especially in the therapeutic setting, we would like her to be the same language mediator”. We also had a situation that involved long-term therapy and initially only one [male] colleague was present, but the client was a lady and it was about sexualised violence and suchlike. And then at some point we switched that and sent our [female] colleague, and then over time you also noticed, okay, now [the client] is opening up […] the language mediator also plays a role, it's not just someone replaceable XY that you can put in there. (Interview16, translation coordinator, Berlin)

As one translator herself emphasises:

[Its] is not about interpreting. We know the culture. […] We know these cultures. I know both cultures […]. The intercultural aspect is very important because we tell stories. (Interview17, professional translator, Berlin)

Whilst the state-funded translation service in Berlin is a pioneer in translation offerings in healthcare and is highly sought after, it is vulnerable to financial shifts. According to interviewees, the service was less readily available at the time of interviewing, as in the months prior. On the one hand, as one gynaecologist and abortion counsellor pointed out, this means that “they are no longer making these spontaneous offers” (Interview05), meaning that if clients arrive without appointments and there is no translator for her language arranged for that day, a translator cannot be telephoned into the consultation on short notice. On the other hand, as the coordinator for the state-funded service in Berlin points out, budget cuts also mean that the pool of translators shrinks due to lay-offs, or reduction in hours, such that the necessary soft-skills and thematic expertise may not always be available:

And because everyone develops differently and then also has their specialties, it is of course also, well, we have many languages and actually we would need an even bigger team to be able to place people more appropriately, so to speak, not just to say, oh yes, we now have ten people for Arabic, so Arabic is fine, it doesn't matter which of the ten people can go there, it's not like that. It really depends on these soft skills. Yes, but if our team gets cut again, unfortunately, then we can't convey it that way anymore. Then we also have to say, okay, we don't have anyone, either this person or [we] can't… (Interview16, translation coordinator, Berlin)

This is a worrying development given the incredible significance of translators in this sector.

In Berlin, the state-funded centralised translation service could provide a best-practice model not just for other federal states like Brandenburg, but also for other national contexts. However, this service is threatened by financial instability and is heavily oversubscribed. High demand means the service receives hundreds of requests a day and is booked sometimes as early as nine months in advance. As a result, some providers are unable to find a translator, and even then, the most appropriate translator is not always available.

Our findings comport with global evidence that demonstrates that migrants and asylum-seekers specifically may lack information about how to access safe abortion in their new settings, as well as local research that demonstrates that the availability of translators is integral to healthcare provision in Germany (Bockey et al., 2024; Erhardt-Ohren and Lewinger, 2020).

## 4 Discussion

This study identified two interrelated categories of barriers for migrant access to abortion care in Germany. First, barriers arise from the current legal frameworks for abortion and migration. Second, barriers are embedded in the healthcare system itself and relate to the lack of diversity among counsellors and healthcare providers, specifically the limited and unequal availability of information and services in the languages of clients. These barriers are mutually reinforcing and cumulatively constraining migrants’ agency and access to timely, dignified abortion care.

### 4.1 Legal and governance-related barriers

Existing literature highlights that mandatory counselling is a barrier to abortion care for all people seeking abortions (Meer et al., 2025, Rowlands and Thomas, 2020; WHO 2022). Our findings complement this literature by showing that, for migrants and asylum-seekers, mandatory counselling may impose an additional and qualitatively distinct burden. Consistent with the recent report by the German Parliamentary Expert Commission on Reproductive Autonomy which recommends the decriminalisation of abortion in the first trimester (2024), our analysis demonstrates that the current regulatory framework exacerbates inequities in access. Notably, the evidence analysed by the commission did not specifically include the perspectives of migrant abortion-seekers.

For migrants and asylum-seekers, interfacing with state agencies is rarely neutral, but forms part of a matrix of governance characterized by the persistent threat of criminalisation or discrimination (Shahvisi and Finnerty, 2019). Within this context, mandatory abortion counselling may be experienced not as supportive, but as another site of scrutiny. Participants described apprehension among migrant abortion-seekers about saying the “wrong” thing to counsellors, jeopardising the mandatory counselling certificate, or even risking adverse consequences for their legal status. Similar fears have been documented for migrants seeking mental healthcare in Germany, and for those seeking abortions in other contexts (Shahvisi and Finnerty, 2019, Mugambwa et al., 2023).

Being subject to more state mandates compounds the erosion of agency and self-worth that result from potential harms of migration and the stigma of reproductive health concerns (Endler et al., 2020). Given the criminalised status of abortion in Germany, this may feel especially acute when accessing abortion-related services.

### 4.2 Discrimination and professional gatekeeping

Our findings show that migrants may be exposed to discriminatory attitudes or practices within mandatory counselling services. Professionals themselves may hold ambivalent or prejudicial views about migrants or consider working with migrants unfavourably compared to working with German women, resulting in poorer-quality or inappropriate services. Evidence shows that migrants and asylum-seekers are especially vulnerable to bureaucratic discrimination (Brussing et al., 2019; Ratzmann, 2022), and that persons who are perceived to be migrants frequently experience racism within healthcare settings in Germany (Rassismus und seine Symptome Bericht des Nationalen Diskriminierungs, 2023).

The mandatory nature of counselling exacerbates these risks. Clients cannot opt out of counselling, and in regions where services are scarce, they often lack meaningful choice of counsellors (Torenz et al., 2023). Importantly, because they are reliant on the counsellor in front of them to receive the certificate that is needed to access an abortion, migrant clients may feel compelled to tolerate discriminatory behaviour to access health care. This vulnerability to discrimination affects not only recent migrants, but anyone perceived as a migrant or a racialised person. This may be especially pronounced where there are few resources and support services for migrants, such as in Brandenburg, and thus little advocacy, mediation or accountability for biased or discriminatory providers.

### 4.3 Structural constraints in collective accommodation settings

Barriers are further intensified for asylum-seekers living in temporary collective accommodation. While there is limited research on health in these settings, the few recent studies found high unmet health needs (Biddle et al., 2021) and poor access to sexual and reproductive healthcare (Inci et al., 2020). Our findings reveal that refugees and asylum seekers in collective accommodations face profound constraints on autonomy, privacy, or freedom of movement, all of which directly affect access to abortion care.

In contrast to Berlin, Brandenburg accommodation facilities are typically located in remote and rural areas, with limited access to healthcare infrastructure. This is compounded by legal restrictions on mobility for the first months of the asylum process, which prohibit leaving the assigned district without permission (Asylgesetz (AsylG) § 56 Räumliche Beschränkung 2024). Asylum-seekers are entitled to free emergency healthcare within the German public health system; however, access requires registration with statutory health insurance first, which can take several months. During the waiting period, individuals can only access emergency healthcare through health insurance vouchers distributed by social workers or welfare offices. This necessitating disclosing their need for abortion to officials, which may be especially difficult given abortion stigma. This system also places a high burden on officials, and lack of knowledge about medical needs coupled with uncertainty about the law can lead to highly variable decision-making (Classen, 2018; Healthcare voucher – Gesundheit für Geflüchtete 2017).

As a result, basic healthcare needs are subject to multiple layers of scrutiny, involving security personnel, social workers, and administrative authorities. Each layer presents potential sites of delay or discrimination. For individuals with restricted mobility and high dependency on institutional actors, this produces a profound lack of self-determination. In matters related to contraception, sexuality, and pregnancy, such conditions constitute a significant infringement of reproductive rights and dignity, with detrimental effects on agency and self-esteem (Endler et al., 2020; Frings, 2017; Wegelin et al., 2023).

### 4.4 Language, information and communication barriers

Consistent with global evidence, our findings demonstrate that migrants and asylum-seekers face significant language and information barriers when seeking abortion care. Complex regulations, combined with the criminalised status of abortion, mean that many migrants may not know whether abortion is legal in Germany or how to access services. We identified a pervasive lack of accurate, plain-language, and easily accessible information throughout the abortion care pathway (Erdman and Johnson, 2018; Kavanaugh et al., 2019). Language barriers were particularly pronounced in abortion counselling.

Counsellors in Brandenburg explicitly highlighted the mismatch between their own racial, ethnic and religious identities and experiences and those of their clients, and appeared to have limited experience, skills, and institutional support for working with diverse groups. These challenges were closely linked to having less access to translators and weaker connections to migrant-specific organisations compared to Berlin, where longer-standing diversity has fostered better support structures and referral networks. Overall, the existing mandatory counselling framework, with its limited linguistic and cultural adaptability, appears poorly aligned with the needs of migrant clients.

Germany-based research underscores that the availability of skilled translators is integral to healthcare provision in Germany (Bockey et al., 2024; Erhardt-Ohren and Lewinger, 2020). Professional interpreting services facilitate more accurate, nuanced, uninhibited, and confidential communication than ad-hoc arrangements, improving both clinical outcomes and patients’ sense of safety and trust (Flores, 2005). According to our findings, professional interpreting services specialising in healthcare issues are underfunded and unavailable, leaving migrant individuals reliant on informal translators whom they must arrange themselves, and who may inflect translation with their own agenda, posing threats to confidentiality (Leanza et al., 2010; Rosenberg et al., 2008). This is particularly problematic in abortion care, where confidentiality and stigma are central concerns.

Access to information and effective communication are integral to dignity in healthcare. It enables informed, independent decision-making, reduces miscommunication about clinically relevant issues, but also aids individuals’ sense of agency that may be undermined by migration-related experiences (Endler et al., 2020). Moreover, good communication can counteract stereotyping by enabling providers to respond to individual needs rather than assumptions. This is especially relevant given the prevalence of stereotypes about migrants within German society and healthcare settings (Chakraverty et al., 2020; FitzGerald and Hurst, 2017; Rassismus und seine Symptome Bericht des Nationalen Diskriminierungs, 2023).

### 4.5 Limitations

Our study was limited to Berlin and Brandenburg, and our findings may therefore not accurately reflect the specificities of abortion barriers for migrants in other federal states in Germany. Furthermore, since we employed snowball sampling, there is potential for selection bias. We only included the perspectives of service providers, translators, and activists instead of migrants who have experienced abortion in Germany. Additional research including the perspective of individuals affected would be extremely valuable. However, as service providers have detailed knowledge of a highly complex system and act as intermediaries between the system and migrant abortion-seekers, we believe that the perspective of service providers offers important insights into the structural barriers to abortion care for migrants in Germany.

### 4.6 Conclusion

Taken together, our findings demonstrate that while migrants experience many of the same barriers as other abortion-seekers, these barriers are intensified by migration-specific constraints, including restricted mobility, limited healthcare entitlements, linguistic exclusion, and heightened exposure to state control. In the context of Germany’s criminalised abortion framework, these factors combine to create a particularly labyrinthine pathway to care, with mandatory counselling playing a central role in exacerbating inequities (Maeffert and Tennhardt, 2023).

Our analysis complements and strengthens the conclusions of the Parliamentary Expert Commission on Reproductive Autonomy (Bericht der Kommission zur reproduktiven Selbstbestimmung und Fortpflanzungsmedizin, 2024). By documenting the disproportionate burdens faced by migrants and asylum-seekers, our findings provide additional support for the Commission’s recommendation to decriminalise abortion in the first trimester. Decriminalisation would reduce procedural complexity, mitigate power imbalances between clients and counsellors, and improve access to care for groups already at heightened risk of unwanted pregnancy.

The contrast between Berlin and Brandenburg further illustrates the importance of structural diversity within healthcare systems. In Berlin, greater racial, ethnic and religious diversity among providers, alongside better integration of language services, showed better access and quality of care for migrants. These differences persist despite Brandenburg hosting more collective temporary accommodation for asylum seekers. While funding and service availability remain critical, our findings suggest that diversity and cultural competence at a structural level meaningfully enhance access to abortion care for migrant populations. Beyond abortion care, our findings underscore the broader need for a healthcare system that reflects the social and linguistic diversity of German society (Mews et al., 2018). This includes strengthening cultural and diversity competence among German-born and German-speaking providers, embedding interpretation services as a standard component of care, and recognising the qualifications of healthcare workers who have migrated to Germany. Persistent barriers to recognising foreign credentials contribute to the underemployment of skilled migrants, despite their linguistic and cultural expertise representing a significant asset to the healthcare system (Damelang et al., 2020).

Finally, decriminalising abortion in the first trimester and improving the integration of migrants into healthcare and allied service provision would free up resources currently devoted to surveillance and regulation. These resources could instead support multilingual information provision, voluntary counselling models, and low-barrier, rights-based approaches that enhance dignity and autonomy for all abortion-seekers, and particularly for migrants and asylum-seekers. (Clasen and Völckel, 2022)

## Funding sources

The study is part of the Grand Challenges Initiative on Global Health- Exploration Project “MigraH”, funded under the Excellence Strategy of the Federal Government and the Länder by the Berlin University Alliance (BUA). The BUA had no input into the conduct and analysis of the study.

## CRediT authorship contribution statement

**Talia Meer:** Writing – review & editing, Writing – original draft, Methodology, Investigation, Formal analysis. **Alex Müller:** Writing – review & editing, Methodology, Investigation, Formal analysis, Conceptualization. **Mascha Kern:** Writing – review & editing, Methodology, Investigation. **Bornice Biomndo:** Writing – review & editing, Methodology, Investigation. **Yagmur Demirpehlivan:** Writing – review & editing, Project administration, Methodology. **Muhammad Helmi Barghouth:** Writing – review & editing, Project administration, Methodology. **Stefanie Theuring:** Writing – review & editing, Methodology, Investigation, Funding acquisition, Formal analysis, Conceptualization.

## Declaration of competing interest

The authors declare that they have no known competing financial interests or personal relationships that could have appeared to influence the work reported in this paper.
